# One year follow-up of a pragmatic multi-centre randomised controlled trial of a group-based fatigue management programme (FACETS) for people with multiple sclerosis

**DOI:** 10.1186/1471-2377-14-109

**Published:** 2014-05-19

**Authors:** Peter W Thomas, Sarah Thomas, Paula Kersten, Rosemary Jones, Vicky Slingsby, Alison Nock, Angela Davies Smith, Roger Baker, Kathleen T Galvin, Charles Hillier

**Affiliations:** 1 Clinical Research Unit, School of Health and Social Care, Bournemouth University, Bournemouth BH1 3LT, UK; 2 Person Centred Research Centre, School of Rehabilitation and Occupation Studies, Auckland University of Technology, Auckland, New Zealand; 3 MS Research Unit, Bristol and Avon MS Clinical Centre, Frenchay Hospital, Bristol BS16 1LE, UK; 4 Dorset MS Service, Poole Hospital NHS Foundation Trust, Poole BH15 2JB, UK; 5 Faculty of Health and Social Care, Hull University, Hull HU6 7RX, UK

**Keywords:** Randomised controlled trial, Multiple sclerosis, Fatigue, Intervention, Energy effectiveness, Cognitive behavioural, Group

## Abstract

**Background:**

Fatigue is one of the most common and debilitating symptoms of multiple sclerosis (MS). The aim was to evaluate the effectiveness at 1-year follow-up of a manualised group-based programme (‘FACETS’) for managing MS-fatigue.

**Methods:**

One-year follow-up of a pragmatic multi-centre randomised controlled trial. People with MS and significant fatigue were randomised to FACETS plus current local practice (FACETS) or current local practice alone (CLP), using concealed computer-generated randomisation. Participant blinding was not possible. Primary outcome measures were fatigue severity (Global Fatigue Severity subscale of the Fatigue Assessment Instrument), self-efficacy (MS-Fatigue Self-Efficacy) and disease-specific quality of life (MS Impact Scale).

**Results:**

Between May 2008 and November 2009, 164 participants were randomised. Primary outcome data were available at 1 year for 131 (80%). The benefits demonstrated at 4-months in the FACETS arm for fatigue severity and self-efficacy largely persisted, with a slight reduction in standardised effect sizes (SES) (−0.29, p = 0.06 and 0.34, p = 0.09, respectively). There was a significant difference on the MS Impact Scale favouring FACETS that had not been present at 4-months (SES −0.24, p = 0.046). No adverse events were reported.

**Conclusions:**

Improvements in fatigue severity and self-efficacy at 4-months follow-up following attendance of FACETS were mostly sustained at 1 year with additional improvements in MS impact. The FACETS programme provides modest long-term benefits to people with MS-fatigue.

**Trial registration:**

ISRCTN76517470

## Background

Fatigue is one of the most common and debilitating symptoms of MS [1-5]. We have developed a non-pharmacological group-based fatigue management programme for people with MS called FACETS (Fatigue: Applying Cognitive behavioural and Energy effectiveness Techniques to lifeStyle) [6]. One of the criticisms of non-pharmacological trials is that they often do not include any long term follow-up. This paper reports on one year follow-up data obtained from a pragmatic three-centre trial of FACETS [7].

## Methods

We carried out a pragmatic multi-centre randomised controlled trial following the published protocol [8] in which the full study design, inclusion and exclusion criteria, trial arms, and plan of analysis are described in detail. The main results are reported elsewhere [7]. Here we report the results from the 1 year self-reported follow-up data.

Participants were recruited in three UK centres (Poole, Bristol, Southampton/Portsmouth) from primary or secondary care, or via MS Society newsletters/websites. Recruitment took place from May 2008 to November 2009. Ethical approval was obtained from the South West-Central Bristol Research Ethics Committee (ref: 08/H0106/2). All participants provided written informed consent before taking part.

The main inclusion criteria were: (1) clinically definite MS diagnosis, (2) fatigue impacting on daily life (Fatigue Severity Scale total score >4) [9] and (3) ambulatory. The main exclusion criteria were: (1) having taken part in a fatigue programme in the last year, (2) cognitive impairments (3) a relapse in the previous 3 months or (4) having started treatment with disease modifying or antidepressant drugs within the previous 3 months. The full eligibility criteria are described in the protocol [8].

### Intervention (FACETS programme)

The manualised group-based FACETS programme is described elsewhere [6] and is based upon a conceptual framework integrating elements from cognitive behavioural, social-cognitive, energy effectiveness, self-management and self-efficacy theories. The aim of the intervention is to help people with MS normalise their fatigue experiences, learn helpful ways of thinking about fatigue and use available energy more effectively. The intervention consists of six sessions (∼90 min duration) held weekly and facilitated in groups of 6–12 by two health professionals with experience of working with people with MS and group-work (such as occupational therapists, nurses or physiotherapists). Each session follows the same general format, namely, facilitator-delivered presentations, flipchart discussions, group activities and homework. The facilitator manual provides guidance on preparation and delivery, detailed session content, notes and suggested timings, and a checklist of facilitator objectives as well as signposts to additional resources. Sessions are delivered via PowerPoint; hence can be easily replicated. A companion participant handbook, along with existing information booklets, reinforces programme content.

FACETS was delivered in hotel meeting-room facilities, with the exception of one centre, where it was held in a rehabilitation hospital. Apart from one MS specialist nurse, facilitators were either occupational therapists or physiotherapists. Facilitators were trained to deliver the intervention at 1-day workshops and psychological advice and debriefing were available for facilitators throughout the trial.

To increase external validity, no attempt was made in the FACETS arm to restrict or control participants’ access to current local practice or to standardise it across healthcare settings or treatment arms. When we refer to the FACETS arm, participants in this arm also received current local practice.

### Control group (Current Local Practice (CLP))

Participants randomised to this arm of the trial received current local practice.

This could have ranged from general advice and information provision about MS-fatigue to more detailed individualised management advice from a variety of health professionals. Inevitably, there will have been variations in the exact composition of what was usually provided, within and between centres, depending on local resources and patient need. Collecting detailed information at an individual level on the type and quantity of advice received as part of current local practice was not deemed feasible. However, this real world variation increases applicability to a wider range of centres.

### Outcomes

For those allocated to the FACETS arm outcomes were measured 1 week (baseline) before the start of the FACETS programme and 1 month (follow-up 1), 4 months (follow-up 2) and 12 months (follow-up 3) after the final session. Participants in the current local practice arm completed outcome measures within an identical time frame. Data from follow-up 1 and 2 have previously been reported [7]. In this paper we focus on reporting follow-up 3.

Primary outcomes were fatigue severity (Global Fatigue Severity (GFS) subscale of the Fatigue Assessment Instrument (FAI)), disease specific quality of life (Multiple Sclerosis Impact Scale (MSIS-29, V.1)) and self-efficacy for managing fatigue (Multiple Sclerosis - Fatigue Self-Efficacy scale (MS-FSE)) [7,8].

Secondary outcomes included the Fatigue Symptom Inventory (FSI), the Hospital Anxiety and Depression Scale (HADS), the Medical Outcomes Short-Form Survey (SF-36, V. 2), and subscales of the MSIS-29 (V.1) and the FAI [7,8]. All outcomes collected at 12 months were self-reported questionnaires and administered postally.

### Sample size considerations

The sample size requirement was 146 participants with follow-up data based on having 85% power to detect a medium standardised effect size of 0.5 for the primary outcome measures, using a two-sided 5% significance level (see protocol for justification for this medium effect size) [8]. As a variety of fatigue measures have been used in other trials, we used standardised effect sizes to enable comparisons between them.

### Analysis

The main analysis was intention-to-treat but we also conducted a per protocol analysis (excluding participants who attended fewer than four FACETS sessions). Data were analysed using IBM SPSS, V.18 and MLwiN 2.17. Outcome measures were assumed to be interval-scaled and the main analysis focused on absolute change in outcomes at 1 year follow-up relative to baseline. Change scores were compared between the groups using the independent samples t-test with a two sided 5% significance level, and summarised using mean differences (95% confidence intervals (CIs)) and standardised effect sizes (SES). As detailed in the protocol, additional pre-specified supplementary analyses were undertaken. Here we report results from a mixed model approach that includes 1 year and baseline measurements as repeated measures, incorporates clustering effects, and includes pre-specified covariates (baseline for other primary outcomes, age, gender, marital status, education level, type of MS, time since diagnosis, level of disability, and centre)*.*

## Results

**Figure 1:**
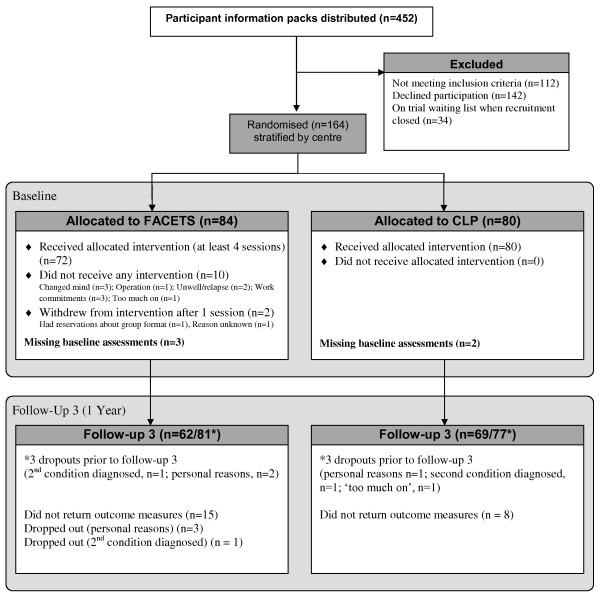
Participant flow.

One year follow up data are available on 131 participants (80%) (Figure 1). The distributions of descriptive statistics for the trial sample are presented in Table 1.

**Table 1:** Descriptive demographic and baseline characteristics of participants

	**FMP (n = 84)**	**CLP (n = 80)**
**Gender [n (%)]**		
▪ Female	61 (73%)	58 (73%)
▪ Male	23 (27%)	22 (28%)
**Age (years)**		
Mean (S.D.)	48.0 (10.2)	50.1 (9.1)
Range	23-73	28-70
**Ethnicity [n (%)]**		
▪ White English	68 (85%)	69 (92%)
▪ White British	7 (9%)	5 (7%)
▪ Other	5 (6%)	1 (1%)
▪ Not stated	4	5
**Disease type (self-reported) [n (%)]**		
▪ Benign	4 (5%)	2 (3%)
▪ Relapsing-remitting	35 (43%)	40 (51%)
▪ Secondary progressive	16 (20%)	23 (29%)
▪ Primary progressive	5 (6%)	8 (10%)
▪ Participant states “Don’t know”	21 (26%)	5 (6%)
▪ Not stated	3	2
**APDDS score (Adapted Patient Determined Disease Steps) [n (%)]**		
▪ 3 or less (No limitations on walking)	18 (22%)	15 (19%)
▪ 4 or 5 (MS interferes with walking)	37 (46%)	42 (54%)
▪ 6 or more (At min., needs stick/crutch to walk 100 m)	26 (32%)	21 (27%)
▪ Not stated	3	2
**Level of education [n (%)]**		
Highest qualification achieved:		
▪ No qualifications	8 (10%)	8 (10%)
▪ One or more GCSE (or equiv.)	36 (46%)	29 (38%)
▪ One or more A level (or equiv.)	10 (13%)	12 (16%)
▪ First degree (or equiv.)	16 (20%)	19 (25%)
▪ Higher degree/professional qualification	9 (11%)	8 (11%)
▪ Not stated	5	4
**Employment status [n (%)]**		
▪ In full time employment (>30 hours per week)	15 (18%)	11 (14%)
▪ In part-time employment (≤30 hours per week)	11 (14%)	13 (17%)
▪ Self-employed	4 (5%)	4 (5%)
▪ Not in paid employment (unemployed, in education, retired, looking after home)	51 (63%)	50 (64%)
▪ Not stated	3	2
**Marital status [n (%)]**		
▪ Married/cohabiting	63 (78%)	54 (71%)
▪ Single	5 (6%)	7 (9%)
▪ Separated/divorced	9 (11%)	14 (18%)
▪ Widowed	4 (5%)	1 (1%)
▪ Not stated	3	4
**Years since diagnosis [n (%)]**		
▪ <1 yr	2 (3%)	4 (5%)
▪ 1–5 yrs	32 (40%)	21 (27%)
▪ 6–10 yrs	13 (16%)	19 (24%)
▪ 11–15 yrs	21 (26%)	12 (15%)
▪ ≥16 yrs	12 (15%)	22 (28%)
▪ Not stated	4	2

Percentages rounded to nearest integer and, thus, might not sum exactly to 100%.

CLP, current local practice; FACETS, Fatigue: Applying Cognitive behavioural and Energy effectiveness Techniques to lifeStyle; GCSE, General Certificate of Secondary Education; MS, multiple sclerosis.

### Primary outcomes

The intention-to-treat analyses and results for the primary outcome measures are shown in Table 2.

**Table 2:** Descriptive statistics and treatment effects at 1 year follow-up

**Primary outcome measures**	**Baseline (n = 159)**	**Follow-up 1 (n = 146)**	**Follow-up 2 (n = 144)**	**Follow-up 3 (n = 131)**
**Global fatigue severity (GFS) subscale of the FAI***(potential range 1 to 7, high scores indicate more fatigue)*				
FACETS mean (SD)	5.60 (0.98)	5.48 (0.92)	5.26 (1.03)	5.32 (1.00)
CLP mean (SD)	5.61 (1.09)	5.55 (1.17)	5.66 (0.93)	5.70 (1.01)
Mean difference in change from baseline [95% CI]	**-**	−0.03 (−0.33 to 0.28)	−0.36 (−0.63 to −0.08)	−0.30 (−0.61 to 0.01)
p value	**-**	0.86	0.01	0.06
Std effect size	**-**	−0.03	−0.35	−0.29
**Multiple Sclerosis Impact Scale-29 (MSIS-29)***(potential range 0 to 100, high scores indicate more impact)*				
FACETS mean (SD)	49.6 (19.1)	47.3 (18.2)	44.9 (19.2)	46.2 (19.1)
CLP mean (SD)	43.9 (17.6)	42.2 (18.4)	43.0 (17.3)	47.2 (17.4)
Mean difference in change from baseline [95% CI]	**-**	1.44 (−2.36 to 5.24)	−1.56 (−6.45 to 3.34)	−4.34 (−8.61 to −0.08)
p value	**-**	0.46	0.53	0.046
Std effect size	**-**	0.08	−0.08	−0.24
**MS Fatigue Self-Efficacy scale (MS-FSE)***(potential range 10 to 100, high scores indicate more certainty in controlling fatigue*				
FACETS mean (SD)	45 (17)	57 (17)	56 (19)	56 (16)
CLP mean (SD)	49 (16)	50 (17)	53 (17)	52 (17)
Mean difference in change from baseline [95% CI]	**-**	9 (4 to 14)	6 (0 to 12)	6 (−1 to 12)
p value	**-**	0.001	0.048	0.09
Std effect size	**-**	0.54	0.36	0.34
**Statistically significant secondary outcome measures**				
**Vitality subscale of the SF-36***(potential range 0 to 100, high scores indicate higher quality of life)*				
FACETS mean (SD)	32.0 (16.8)	35.6 (19.4)	37.4 (20.3)	37.70 (18.75)
CLP mean (SD)	35.1 (19.7)	33.4 (16.8)	34.4 (17.30	32.43 (17.69)
Mean difference in change from baseline [95% CI]	-	4.42 (−1.22 to 10.06)	6.38 (0.45 to 12.32)	6.64 (0.84 to 12.44)
p value	**-**	0.12	0.04	0.03
Std effect size	-	0.24	0.35	0.37
**Multiple Sclerosis Impact Scale-29 (MSIS-29) - Physical subscale***(potential range 0 to100, high scores indicate more impact)*				
FACETS mean (SD)	51.4 (21.4)	48.8 (19.7)	47.0 (21.3)	47.4 (21.0)
CLP mean (SD)	46.6 (20.3)	44.9 (20.5)	46.5 (19.8)	50.5 (20.1)
Mean difference in change from baseline [95% CI]	-	1.39 (−2.87 to 5.65)	−0.81 (−5.91 to 4.28)	−4.74 (−9.40 to −0.08)
p value	**-**	0.52	0.75	0.046
Std effect size	-	0.07	−0.04	−0.23

Results for fatigue severity and self-efficacy were similar to those at 4 months with a slight reduction in standardised effect size (SES) from −0.35 (p = 0.01) for fatigue severity to −0.29 (p = 0.06) and from 0.36 (p = 0.048) for fatigue self-efficacy to 0.34 (p = 0.09). There were significantly greater improvements on the MSIS-29 for the FACETS arm compared with the CLP arm (p = 0.046, SES = −0.24) that were not evident at 4 months.

The per protocol analysis resulted in an increased SES for the MSIS-29 (from −0.24 to −0.26 (p = 0.03)) and for the MS-FSE (from 0.34 to 0.39 (p = 0.046)). The SES for the GFS subscale was reduced from −0.29 to −0.25 (p = 0.10).

Participants in the FACETS arm were 1.5 times more likely (31% (19/62) versus 20% (14/69)) to have a clinically important improvement on the GFS (defined as an individual reduction of ≥ 0.5), although, unlike at 4 months, this was not statistically significant (p = 0.25 using chi-squared test with continuity correction).

Using the mixed model approach, the mean difference at 1 year for GFS was almost unchanged (−0.28 (−0.58, 0.02), p = 0.07), for fatigue self-efficacy was slightly higher (7 (1, 13), p = 0.02), and for the MSIS-29 slightly lower (−3.90 (−8.08, 0.28), p = 0.07).

### Adverse events

No adverse events, as defined in the protocol, were reported.

### Secondary outcomes

For the MSIS-29 physical subscale (p = 0.046, SES = −0.23) and the vitality subscale of the SF-36 (p = 0.03, SES = 0.37) there was a significant difference in favour of the FACETS arm at 1 year. None of the other secondary outcomes was statistically significant. Effect sizes and significance levels were similar using the mixed model approach.

## Discussion

The modest improvements in fatigue severity and fatigue self-efficacy in the FACETS arm at 4 months were largely maintained at 1 year. While attrition was relatively low at one year there was a diminution of sample size to 131 (the original sample size calculation requirement was n = 146). Statistical power would have been reduced slightly to 80% (NQuery Advisor) and while still a reasonable level of power this is lower than that at follow-ups 1 and 2 and might account in part for the slightly larger p-values obtained at the 1 year follow-up.

In addition to improvements in fatigue severity and self-efficacy there were improvements in MS-specific quality of life that had not been present at 4 months follow-up. The delayed appearance of this latter impact might be because the changes to lifestyle encouraged by the FACETS programme may take some time to implement effectively.

Only some of those who declined participation in the FACETS trial provided reasons for doing so. When reasons were provided, they were predominantly related to lack of time or existing work, holiday or childcare commitments. A small minority of individuals felt that a group approach was not for them or did not wish to take part in a research trial. However, we acknowledge it is possible that there might have been a recruitment bias towards those more amenable to a non-pharmacological approach.

## Conclusions

FACETS appears to have long term benefits for people with MS at an estimated cost of £453 per person. Often trials of nonpharmacological interventions do not measure long term follow-up or effects do not persist beyond the short term. Given the progressive nature of MS and the debilitating nature of fatigue, our demonstration of small to medium improvements at 1 year follow-up is encouraging.

## Competing interests

All authors had financial support from the Multiple Sclerosis Society in the UK for the submitted work; no financial relationships with any organisations that might have an interest in the submitted work in the previous three years. RB is the chair of the MS Society Grant Review Panel for Care and Services Research. PT is a member of the MS Society Grant Review Panel for Care and Services Research. PT is a member of the Advisory Board for the Sativex Registry. The Board provides an independent review of safety data for patients prescribed Sativex. Bournemouth University receives a fee from GW Pharma to cover time spent at meetings, and travel expenses.

## Authors’ contributions

Contributors: PT: chief investigator, conception, design, analysis, interpretation, drafted article. ST: conception, design, acquisition of data, analysis, interpretation, drafted article. PK and RJ: design, acquisition of data, interpretation, critically reviewed article. AN and VS: design, delivered fatigue management programme, acquisition of data, interpretation, critically reviewed article. ADS: design, delivered fatigue management programme, critically reviewed article. RB and KTG: design, interpretation, critically reviewed article. CH: design, clinical oversight of trial, interpretation, critically reviewed article. All authors read and approved the final manuscript.

## Pre-publication history

The pre-publication history for this paper can be accessed here:

http://www.biomedcentral.com/1471-2377/14/109/prepub
